# Lipidome analysis of rotavirus-infected cells confirms the close interaction of lipid droplets with viroplasms

**DOI:** 10.1099/vir.0.049635-0

**Published:** 2013-07

**Authors:** Eleanor R. Gaunt, Qifeng Zhang, Winsome Cheung, Michael J. O. Wakelam, Andrew M. L. Lever, Ulrich Desselberger

**Affiliations:** 1 Department of Medicine, University of Cambridge, Addenbrooke’s Hospital, Cambridge CB2 0QQ, UK; 2 The Babraham Institute, Babraham Research Campus, Cambridge CB22 3AT, UK

## Abstract

Rotaviruses (RVs) cause acute gastroenteritis in infants and young children, and are globally distributed. Within the infected host cell, RVs establish replication complexes in viroplasms (‘viral factories’) to which lipid droplet organelles are recruited. To further understand this recently discovered phenomenon, the lipidomes of RV-infected and uninfected MA104 cells were investigated. Cell lysates were subjected to equilibrium ultracentrifugation through iodixanol gradients. Fourteen different classes of lipids were differentiated by mass spectrometry. The concentrations of virtually all lipids were elevated in RV-infected cells. Fractions of low density (1.11–1.15 g ml^−1^), in which peaks of the RV dsRNA genome and lipid droplet- and viroplasm-associated proteins were observed, contained increased amounts of lipids typically found concentrated in the cellular organelle lipid droplets, confirming the close interaction of lipid droplets with viroplasms. A decrease in the ratio of the amounts of surface to internal components of lipid droplets upon RV infection suggested that the lipid droplet–viroplasm complexes became enlarged.

## Introduction

Rotaviruses (RVs) form a genus of the family *Reoviridae* and cause significant morbidity and mortality in infants and young children, particularly in the developing world (Estes & Kapikian, 2007; Tate *et al.*, 2012).

RVs possess a genome of 11 segments of ds RNA. Upon entry into cells, RVs lose their outer protein layer, and the resulting double-layered particles (DLPs) immediately start to transcribe ssRNAs of positive polarity, which are released into the cytoplasm where they are translated. Two virus-encoded non-structural proteins (NSPs), NSP2 and NSP5, are necessary for the formation of the cytoplasmic inclusion bodies known as ‘viroplasms’ (Fabbretti *et al.*, 1999). In viroplasms, viral core particles containing proteins VP1, VP2, VP3 and viral RNAs are formed and viral RNA replication takes place. VP6 is acquired in the viroplasm leading to the formation of DLPs which are released into the cytoplasm. DLPs then acquire the outer proteins VP7 and VP4 to become infectious triple-layered particles (TLPs) which are released by cell lysis or a budding process (Estes & Kapikian, 2007).

It was recently reported that RV viroplasms recruit lipid droplets (LDs) during viral replication (Cheung *et al.*, 2010). LDs are cellular organelles acting as repositories for cholesterol (CH) and neutral fats, particularly triacylglycerols (TAGs) and cholesterol esters (CEs), and over 100 protein species are incorporated into their single-layered phospholipid membrane. The LD morphology and composition are highly dynamic (Guo *et al.*, 2009; Martin & Parton, 2006). LDs have heterogeneous proteomic profiles (Ducharme & Bickel, 2008), acquire TAGs at variable rates and the composition of their phospholipid monolayer is flexible (Kuerschner *et al.*, 2008). The proteome of LDs includes over 100 members, many of which contain a conserved structural transmembrane motif and are defined as ‘PAT’ family proteins [archetypal family members are perilipin, adipose differentiation-related protein (ADRP) and TIP-47] (Wolins *et al.*, 2001). Membrane curvature of LDs is dictated by the integration of conical lipids, e.g. lysophosphatidylcholine (LPC)(Suzuki *et al.*, 2011). In Chinese hamster ovary (CHO) cells, the LD surface is enriched in phosphatidylcholine (PC), but depleted of sphingomyelin (SM) and phosphatidylethanolamine (PE) (Bartz *et al.*, 2007). Further, certain species of PC and LPC are upregulated in LD membranes in HepG2 cells (Tauchi-Sato *et al.*, 2002). Members of the family *Flaviviridae*, including hepatitis C virus (HCV), GB virus C, dengue virus and West Nile virus, have also been reported to recruit LDs to replication complexes (Barba *et al.*, 1997; Samsa *et al.*, 2009).

It was previously shown that viral genomic dsRNA, viral proteins NSP2 and NSP5 (as markers of viroplasms) and perilipin (as a marker of LDs) co-migrate at low density in iodixanol gradients of RV-infected cell lysates (Cheung *et al.*, 2010). Whilst the close proximity of NSP5 with perilipin has been demonstrated by Foerster resonance energy transfer analysis (Cheung *et al.*, 2010), relationships between RV viroplasms and other LD components have not been investigated further. Key questions include whether RV infection affects the composition of LDs and whether an aggregation of LDs occurs in synchrony with the fusion of viroplasms described for the late RV replication cycle (Eichwald *et al.*, 2004). To address these questions, the lipidome of RV-infected cell lysates in gradient fractions of different densities was determined by mass spectrometry and compared with that of uninfected cells.

## Results

### Selection of fractions for analytic evaluation

Analysis of the lipidome of RV-infected cells was, in the first instance, undertaken without anti-mitochondrial antibody treatment. A significant increase in the total lipid content of RV-infected cells compared with their uninfected counterparts was observed (details not shown). Two additional sets of paired samples were subsequently fractionated, including samples treated with anti-mitochondrial antibody. The results of these analyses are presented.

**Fig. 1.**
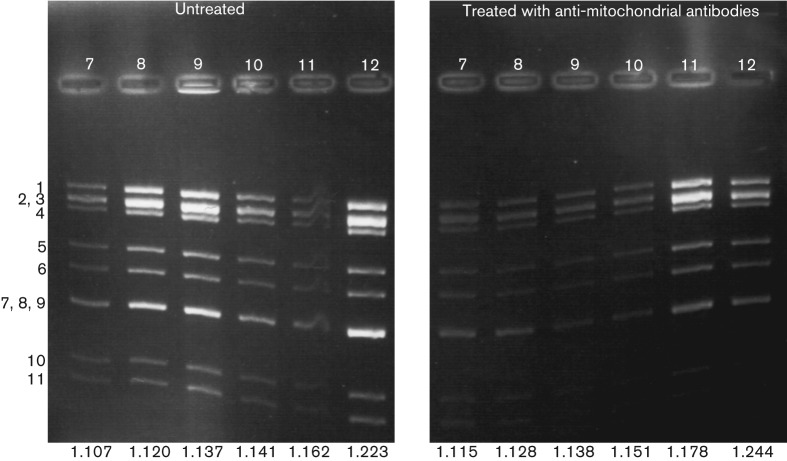
Agarose gel of RV-infected cell lysate fractions 7–12. Densities of fractions (g ml^−1^) are indicated at the bottom of each lane. RV RNA segment numbers are indicated to the left of the image (Kalica *et al.*, 1978).

Gradient fractions from the latter two experiments were tested for the presence of viral dsRNAs (Fig. 1), which were previously shown to co-fractionate with NSP2/NSP5 (components of viroplasms) and with perilipin A (component of LDs) (Cheung *et al.*, 2010). In all, 14 lipid classes were quantified by mass spectrometry [Table 1; Appendix S1 (available in JGV Online); data for individual fractions available upon request].

**Table 1.** Total lipid content by species in RV-infected vs uninfected cells Unin, Uninfected cells; Unin(ab), uninfected cell lysate treated with anti-mitochondrial antibodies; In, infected cells; In(ab), infected cells treated with anti-mitochondrial antibodies. Cer, Ceramide; CL, cardiolipin; FFA, free fatty acids; LPC, lysophosphatidylcholine; PA, phosphatidic acid; PC, phosphatidylcholine; PE, phosphatidylethanolamine; PG, phosphatidylglycerol; PI, phosphatidylinositol; PS, phosphatidylserine; SM, sphingomyelin; TAG, triacylglycerol; CE, cholesterol ester; CH, cholesterol.

Lipid class	Total detected (ng per fraction)				Total lipid (%)*			
	Unin	In	Unin(ab)	In(ab)	Unin	In	Unin(ab)	In(ab)
**Experiment 1**								
Cer	389.1	590.4	156.1	256.9	1.82	2.31	0.92	1.07
CL	628.3	1 398.9	310.8	423.2	2.93	5.47	1.83	1.76
FFA	4 512.0	6 120.9	4 727.3	5 639.4	21.06	23.94	27.88	23.40
LPC	125.4	67.3	31.2	63.1	0.59	0.26	0.18	0.26
PA	68.4	93.4	74.6	84.8	0.32	0.37	0.44	0.35
PC	3 686.5	3 057.0	2 623.6	3 573.2	17.21	11.96	15.48	14.83
PE	1 373.2	1 258.3	1 468.7	2 524.8	6.41	4.92	8.66	10.48
PG	45.6	112.4	16.7	23.5	0.21	0.44	0.10	0.10
PI	3 441.3	3 781.0	2 567.3	3 612.2	16.07	14.79	15.14	14.99
PS	1 170.4	1 451.8	913.5	1 361.7	5.46	5.68	5.39	5.65
SM	663.1	875.5	480.2	758.3	3.10	3.42	2.83	3.15
TAG	296.7	398.5	137.1	137.0	1.39	1.56	0.81	0.57
CE	802.6	853.3	644.2	951.3	3.75	3.34	3.80	3.95
CH	4 217.4	5 508.1	2 801.6	4 685.4	19.69	21.54	16.53	19.45
**Total**	**21 420**	**25 567**	**16 953**	**24 095**	**100**	**100**	**100**	**100**
**Experiment 2**								
Cer	483.0	529.6	320.5	362.8	1.11	1.24	0.95	0.95
CL	3 801.9	2 478.3	2 188.5	1 814.7	8.76	5.80	6.46	4.73
FFA	6 750.2	9 310.3	5 932.9	8 821.5	15.55	21.78	17.50	23.01
LPC	178.1	209.9	83.1	90.3	0.41	0.49	0.25	0.24
PA	127.2	205.6	89.9	161.5	0.29	0.48	0.27	0.42
PC	4 528.2	4 323.4	4 136.1	5 201.9	10.43	10.11	12.20	13.57
PE	3 013.2	2 792.0	1 962.0	2 335.0	6.94	6.53	5.79	6.09
PG	36.7	29.4	41.9	24.4	0.08	0.07	0.12	0.06
PI	9 250.4	8 844.2	6 633.1	7 550.3	21.31	20.69	19.57	19.70
PS	2 867.6	2 801.3	2 322.4	2 729.4	6.61	6.55	6.85	7.12
SM	1 729.6	1 870.9	1 222.2	1 232.4	3.98	4.38	3.61	3.22
TAG	315.3	214.4	305.5	295.9	0.73	0.50	0.90	0.77
CE	1 856.1	1 803.9	1 580.0	1 534.9	4.28	4.22	4.66	4.00
CH	8 469.5	7 338.3	7 081.9	6 176.5	19.51	17.16	20.89	16.11
**Total**	**43 407**	**42 752**	**33 900**	**38 331**	**100**	**100**	**100**	**100**

* Each lipid class expressed as a percentage of the total amount of lipid detected.

**Fig. 2.**
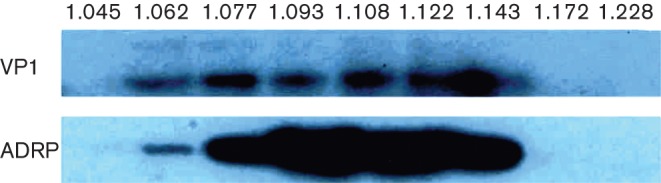
Western blot of subcellular fractions for detection of VP1 (125 kDa) and ADRP (48 kDa). Densities of fractions (g ml^−1^) are indicated at the head of each lane.

The co-fractionation of LD and viroplasm components was further confirmed by Western blotting (Fig. 2). VP1 and ADRP were found to be at highest abundance in the same fractions, which had densities corresponding to those of highest RNA abundance (Fig. 1) and of perilipin A and NSP5 (Cheung *et al.*, 2010).

### Changes of the lipid content of RV-infected cells

In untreated cell extracts, significant increases upon RV infection were recorded for ceramide (Cer), free fatty acids (FFAs), phosphatidic acid (PA) and SM (Table 2), and in anti-mitochondrial antibody-treated cell extracts for Cer, FFAs, LPC, PA, PC, PE, phosphatidylinositol (PI), phosphatidylserine (PS) and SM (Table 2).

**Table 2.** Percentage increase in each lipid class during RV infection

Lipid class	Experiment 1		Experiment 2		Mean post-infection as % of non-infection AM±sem*	
	Post-infection as % of non-infection		Post-infection as % of non-infection			
	Untreated	Antibody treated	Untreated	Antibody treated	Untreated	Antibody treated
Cer	151.7	164.6	109.7	113.2	**130.7±20.0**	**138.9±26.3**
CL	222.6	136.2	65.2	82.9	143.9±78.3	109.5±27.7
FFA	135.7	119.3	137.9	148.7	**136.8±1.1**	**134.0±14.7**
LPC	53.7	202.3	117.9	108.6	85.8±32.1	**155.5±46.2**
PA	136.4	113.7	161.7	179.7	**149.0±12.7**	**146.7±33.0**
PC	82.9	136.2	95.5	125.8	89.2±7.3	**131.0±5.4**
PE	91.6	171.9	92.7	119.0	92.1±0.6	**145.5±26.4**
PI	109.9	140.7	95.6	113.8	102.7±7.2	**127.3±13.4**
PG	246.8	140.9	80.2	58.1	163.5±83.3	99.5±41.4
PS	124.0	149.1	97.7	117.5	110.9±13.1	**133.3±15.8**
SM	132.0	157.9	108.2	100.8	**120.1±11.9**	**129.4±28.8**
TAG	134.3	99.9	68.0	96.9	101.2±33.1	98.4±1.5
CE	106.3	147.7	97.2	97.2	101.8±4.6	121.8±25.9
CH	130.6	167.2	86.6	87.2	108.6±22.0	127.7±40.0
**Total**	**119.4**	**142.1**	**98.5**	**113.1**	**109.0**	**128.5**

* AM, Arithmetic mean; signficant increases are indicated in bold.

In cell extracts treated with anti-mitochondrial antibodies, the greatest enrichments of LD constituents were observed for the fractions of densities of approximately 1.12–1.15 g ml^−1^ for both experimental runs (Tables 3 and 4). Apart from increases in Cer, SM, triglycerides and PI, there was also a remarkable peak for PA, the direct precursor of TAG, in the peak fractions containing RV dsRNA (Tables 3 and 4), strongly suggesting that the synthesis/breakdown of major components of LDs had been activated. It is perhaps surprising that the total amounts of TAGs and CEs were not significantly increased after RV infection (Table 1). This is probably due to the MA104 cell line having a limited number of very small LDs in comparison with adipocytes, liver and other cells (Cheung *et al.*, 2010), agreeing with the observation that the TAGs constitute only 05.–1.5 % and the CEs only 3.5–4.5 % of the total cellular lipids (Table 1). Thus, the analysis of TAGs and CEs will concentrate on the fractions containing peaks of RV dsRNA, viroplasm-associated proteins and LD-associated proteins (see below and Tables 3 and 4).

**Table 3.** Ratio of infected : uninfected of the major lipid droplet components in corresponding fractions of cell lysate (experiment 1) Ratios are given for untreated fractions and fractions which were treated with anti-mitochondrial antibodies (ab). Arithmetic mean±SEM values are calculated for two (three for PA) most abundant species in a lipid class. Most abundant species used for calculations: Cer: 16 : 0, 24 : 1; SM: 16 : 0, 18 : 0; TAG 50 : 1, 52 : 2; PI: 36 : 2, 38 : 2; PA: 34 : 1, 36 : 2, 36 : 1; CL: 68 : 4, 70 : 4 (Appendix S1). Highest ratios in fractions are indicated in bold.

Experiment 1 lipid class	Fraction							
	4	5	6	7	4(ab)	5(ab)	6(ab)	7(ab)
Cer	0.87±0.11	2.37±1.00	**4.10±2.04**	0.17±0.17	1.48±0.2	1.22±0.14	**2.67±0.57**	2.67±0.67
SM	0.45±0.03	**2.00±0.05**	1.38±0.09	0.23±0.20	0.92±0.03	1.46±0.06	**3.21±0.16**	2.20±0.27
TAG	0.09±0	1.27±0.01	**2.38±0.35**	0.30±0.16	0.39±0.03	1.00±0.04	2.12±0.08	**2.43±0.32**
PI	0.28±0.01	**1.41±0**	1.38±0.03	0.71±0.01	0.73±0.03	1.17±0.03	**2.51±0.01**	1.85±0.05
PA	0.40±0.14	**2.29±0.18**	1.17±0.06	0.17±0.03	0.97±0.78	1.69±0.75	**2.86±1.59**	2.06±0.43
CL	0	3.38±0.24	4.2±2.2	–	–	1.22±0.12	1.30±0.14	–
Density (uninf)	1.093	1.117	1.146	1.176	1.086	1.120	1.151	1.184
Density (inf)	1.072	1.113	1.150	1.177	1.068	1.110	1.132	1.179

**Table 4.** Ratio of infected : uninfected of the major lipid droplet components in corresponding fractions of cell lysate (experiment 2) Ratios are given for untreated fractions and fractions which were treated with anti-mitochondrial antibodies (ab). Arithmetic mean±sem values are calculated for the two (three for PA) most abundant species in a lipid class. Most abundant species used for calculations: Cer: 16 : 0, 24 : 1; SM: 16 : 0, 18 : 0; TAG 50 : 1, 52 : 2; PI: 36 : 2, 38 : 2; PA: 34 : 1, 36 : 2, 36 : 1; CL: 68 : 4, 70 : 4 (Appendix S1).

Experiment 2 lipid class	Fraction							
	6	7	8	9	6(ab)	7(ab)	8(ab)	9(ab)
Cer	0.80±0.01	0.71±0.11	**1.31±0.11**	0.40±0.13	0.86±0.03	0.87±0.05	**1.62±0.05**	1.17±0.13
SM	0.92±0.01	0.80±0.02	**1.19±0.13**	0.52±0.02	0.79±0.03	0.83±0.04	**1.89±0.08**	1.78±0.46
TAG	0.62±0.01	0.78±0.05	**1.29±0.10**	0.56±0.04	0.65±0.04	1.87±0.13	**2.09±0.13**	1.12±0.12
PI	0.53±0.02	0.57±0.03	**1.29±0.01**	0.30±0.01	0.69±0.03	0.89±0.03	**2.20±0.10**	1.12±0.03
PA	0.91±0.24	0.88±0.31	**1.54±0.66**	0.97±0.43	0.96±0.22	0.76±0.39	**4.45±3.80**	3.98±2.22
CL	0.13±0.0	0.28±0.01	1.21±0.01	0.32±0.01	0.27±0.01	0.60±0.07	0.70±0.10	1.64±0.01
Density (uninf)	1.094	1.107	1.112	1.138	1.098	1.112	1.125	1.140
Density (inf)	1.094	1.107	1.120	1.137	1.103	1.115	1.128	1.138

The total amount of lipids in infected cells was increased compared with the uninfected control for both untreated lysates and lysates treated with anti-mitochondrial antibodies (Tables 1 and 2). The most abundant lipids in the cells investigated were FFA, PC, PI and CH, with over 70 % of the total cellular lipids accounted for by these four groups alone. Mean increases of lipids in RV-infected cells compared with uninfected cells were calculated (Table 2). The percentage increase of individual lipids in the RV-infected cell extracts compared with uninfected controls was greater in the fractions of cell extracts treated with anti-mitochondrial antibodies (Table 2) [except for phosphatidylglycerol (PG), which was the least detected lipid, and cardiolipin (CL), a major component of mitochondria]. This finding suggests a relative enrichment of LD components after partial removal of mitochondria from the cell extracts before fractionation.

The gradient fractions exhibiting the most significant increase in lipids had densities of 1.11–1.15 (Tables 3 and 4). Mitochondrial densities are in the range 1.16–1.21 (Pollak & Munn, 1970) and so may in part co-fractionate with LDs. The depletion of mitochondria, verified by decreased amounts of the mitochondria-specific lipid CL (Table 1), reduced the potentially confounding inclusion of mitochondrial lipids in these analyses.

The lipid content was analysed further in the fractions containing peak concentrations of RV dsRNA. The density ranges of fractions selected for analysis were 1.068–1.184 (experiment 1) and 1.094–1.140 (experiment 2) (Tables 3 and 4), and the fractions of dsRNA peaks had densities of 1.113–1.151 (experiment 1) and 1.112–1.128 (experiment 2) (Tables 3 and 4). Ratios of concentrations of the major lipid components of LDs in fractions of infected/uninfected cell lysates were determined.

CL levels were measured to assess the success of anti-mitochondrial antibody treatment, since CL is exclusively found in mammalian cell mitochondria. The amounts of CL decreased in uninfected and infected cells following treatment with anti-mitochondrial antibodies (Table 1, experiment 1: uninfected cells had 49.4 % less CL on treatment; RV-infected cells 69.7 %; experiment 2: uninfected cells 57.5 % and RV-infected cells 73.2 %). The higher rate of removal of mitochondria from the samples of experiment 1 is probably due to the lower absolute amount of CL present in samples of experiment 1.

### Increase in size of LDs during RV infection

LDs comprise a hydrophobic core of neutral lipids (triacylglycerides, CEs) and CH, compartmentalized from the aqueous phase of the cell by a phospholipid monolayer (Bartz *et al.*, 2007). Assuming that LDs and viroplasms are nearly perfect spheres, the surface area : volume ratio (SA/V) is inversely proportional to the radius (r) (by a factor of 3/r). We concentrated our analysis on the fractions containing the peak concentrations of dsRNA and LD components, i.e. fractions 5 and 6 of gradient experiment 1 and fractions 7–9 of gradient experiment 2. The sum of (TAG+CE+ CH) was taken as an indicator for LD/viroplasm volume and the sum of the phospholipids as an indicator of the surface. PC and LPC are known to be inserted into the phospholipid monolayer of the LD surface (Tauchi-Sato *et al.*, 2002), and in Huh-7 cells PC and PA are the main components of the LD monolayer, whereas other phospholipids are isolated in lower amounts (Q. Zhang & M. J. O. Wakelam, unpublished data). Overall, it was considered reasonable to include all phospholipids in SA/V calculations. In three out of four gradients the SA/V ratio (Table 5a, b) decreased by 4.0–20.2 % (arithmetic mean±sd: 12.2±8.1 %), potentially reflecting an increase in LD size at 6–8 h post-RV infection, as recorded by Eichwald *et al.* (2004) and seen for LDs in a time-course of RV infection (Cheung *et al.*, 2010). However, during anti-mitochondrial antibody treatment in the second experiment, there was an increase in the SA/V ratio of 30.2 %. This may reflect damage to the organelles which occurred during sample preparation causing a breakdown of the larger LD–viroplasm complexes (see also Fig. 1).

**Table 5.** Lipid components (in ng) in gradient fractions containing peak amount of RV dsRNAs

(a) Experiment 1: fractions 5+6				
	Untreated		Antibody treated*	
Lipid class	Uninfected	Infected	Uninfected	Infected
Cer	226	470	116	191
LPC	109	63	24	51
PA	41	84	40	69
PC	2220	2390	1749	2643
PE	752	1034	1060	2084
PG	31	108	14	21
PI	2296	3419	1962	3072
PS	704	1230	654	1106
SM	395	763	327	607
Subtotal of phospholipids	6774	9561	5946	9844
TAG	156	352	84	111
CE	477	692	416	730
CH	2540	4588	1835	3569
Subtotal of TAG+CE+CH	3173	5632	2335	3569
Ratio of subtotals†	2.13	1.70	2.55	2.23

(b) Experiment 2: fractions 7–9				
	Untreated		Antibody treated*	
Lipid class	Uninfected	Infected	Uninfected	Infected
Cer	263	227	150	184
LPC	72	76	29	41
PA	62	79	34	109
PC	2 007	1 851	1 724	2 649
PE	1 560	864	1 441	1 278
PG	20	14	23	18
PI	5 781	4 297	3 979	5 355
PS	1 430	1 228	1 009	1 575
SM	672	587	335	403
Subtotal of phospholipids	10 867	8 223	8 724	11 612
TAG	153	130	107	174
CE	694	477	643	694
CH	3 440	2 780	2 425	2 589
Subtotal of TAG+CE+CH	4 287	3 387	3 375	3 457
Ratio of subtotals†	2.53	2.43	2.58	3.36

* Anti-mitochondrial antibody treated.

† [Subtotal of phospholipids/subtotal of (TAG+CE+CH)].

### No change to the composition of LDs after RV infection

In order to determine whether the internal composition of LDs changes after RV infection, the CE : TAG ratios were investigated in comparison with uninfected cells. No significant difference was found (Table 6).

**Table 6.** Ratio of CE : TAG in RV-infected and uninfected cells, without and with anti-mitochondrial antibody treatment Means were not significantly different (*t*-test, *P*>0.05). Exp., Experiments.

	CE : TAG ratio						
	Untreated			Antibody treated			Overall
Infection status	Exp. 1	Exp. 2	Mean±sem	Exp. 1	Exp. 2	Mean±sem	Mean±sem
**–**	0.33	0.22	0.28±0.06	0.20	0.17	0.19±0.02	0.23±0.07
**+**	0.51	0.27	0.39±0.12	0.15	0.25	0.20±0.05	0.30±0.15

To seek evidence of desaturase and/or elongase enzyme up- or downregulation during RV infection, the carbon chain length and saturation states of the lipids investigated were assessed. There was no increase in the relative proportion of any specific lipid species relative to other lipid species of a particular group (Appendix S1). This suggests that the relative increase in each lipid class is contributed to by each member species proportionally, and the activities of lipid saturases and elongases are unlikely to be affected by RV infection.

## Discussion

During RV infection, LDs colocalize with viroplasms (Cheung *et al.*, 2010). To explore the impact of RV infection on the LD phenotype, we have characterized the lipidomes of RV-infected cells in comparison with those of uninfected cells. Two major results were apparent. First, the total lipid content of RV-infected cells was significantly higher than that of uninfected cells, reflecting enrichment of LD components. Similar observations have been made by Kim & Chang (2011). The finding that total TAGs and CEs were not significantly increased in RV-infected cells is probably due to the fact that in the cell line used in these experiments (MA104, embryonic monkey kidney-derived) the LDs are only very small and limited in number (Cheung *et al.*, 2010), and thus TAGs and CEs represented only a very minor component of total cellular lipids. The levels of total PA and PG were also very low (Table 1) whilst the levels of di- and mono-acylglycerides were below the level of detection (not shown). Similar findings were obtained for the lipidome of Huh-7 cells after infection with HCV (J. McLauchlan, Q. Zhang, M. Wakelam, unpublished data ). Most data in the literature on LD components have been obtained from adipocytes and macrophages where LDs constitute a much larger percentage of total cellular lipids (Walther & Farese, 2012). In the context of this investigation it will be of interest to investigate the lipidome of Caco-2 cells, a human colon carcinoma-derived cell line which is polarized, gut derived and infectable with RV (Cheung *et al.*, 2010). Secondly, the fractions exhibiting peaks of lipid content also contained peaks of viral dsRNA, the LD-specific protein ADRP and VP1, found in viral core particles and DLPs. Peaks of LD-specific protein perilipin A have previously been shown to accumulate in these fractions (Cheung *et al.*, 2010). In these peak fractions, TAGs and other LD-associated components (Cer, SM, PI, PA) were also shown to peak compared with uninfected cells (Tables 3 and 4). Taken together, these findings support a substantial and striking association of LDs with viroplasms during RV replication and suggest a key role for LDs in RV biogenesis.

Gradient ultracentrifugation of uninfected cell extracts spiked with RV DLPs resulted in the migration of the free DLPs to the bottom of the gradients (Cheung *et al.*, 2010), as expected since rotavirus TLPs, DLPs and cores have densities of 1.36, 1.38 and 1.44 g cm^−3^, respectively (Bican *et al.*, 1982; Chen & Ramig, 1992; Helmberger-Jones & Patton, 1986; Tam *et al.*, 1976). However, in gradients of RV-infected cell lysate, peaks of RV dsRNA, of LD-specific proteins perilipin and ADRP and viral proteins NSP5 and VP1 were seen at much lower densities (1.11–1.15) (this publication and in Cheung *et al.*, 2010). These data therefore reinforce the conclusion that the physical (direct or indirect) association of RV dsRNA with LDs accounts for the low density of the strongest signal of dsRNA bands. By comparison, HCV populations have buoyant densities between 1.06 and 1.17 g ml^−1^ (Hijikata *et al.*, 1993; Kanto *et al.*, 1994; Nielsen *et al.*, 2006). Fractions of this density range contain free enveloped virus particles, as well as HCV particles complexed with high density lipoprotein, antibody, or lipid and antibody (Agnello *et al.*, 1999; Hijikata *et al.*, 1993; Kanto *et al.*, 1994; Monazahian *et al.*, 2000; Nielsen *et al.*, 2006).

The lipids most increased following RV infection were Cer, FFAs, PA and PS. Cers are involved in a range of cellular processes including apoptosis regulation and protein secretion (Bollinger *et al.*, 2005). Rhinovirus and Sindbis virus activate sphingomyelinase resulting in the production of Cer during virus infection (Grassmé *et al.*, 2005) and inducing apoptosis (Jan *et al.*, 2000). Whether RV also activates sphingomyelinase is at present unknown.

PA is associated with the formation of ‘supersized’ lipid droplets; yeast mutants generating enlarged LDs also produced significantly more PA compared with WT, and it is suggested that PA facilitates the coalescence of apposed LDs (Fei *et al.*, 2011). The data reported here similarly show an increase in cellular PA levels correlated with an increase in LD biomass, supporting the possibility that LDs increase in size due to LD fusion events during RV infection. Phospholipase D (PLD) hydrolyses PC to yield PA, and increased expression of PLD1 has been shown to upregulate LD formation (Andersson *et al.*, 2006). The effects of PLD up-/downregulation on RV replication are unknown and remain to be explored, although there are alternative routes of PA formation such as those catalysed by diacylglycerol kinase and lysophosphatidate acyltransferase.

PS is upregulated during infection with HIV-1 human immunodeficiency virus type 1 (Brügger *et al.*, 2006), vesicular stomatitis virus and Semliki forest virus (Kalvodova *et al.*, 2009). It is speculated that bulkier PS species are upregulated by the latter two viruses to increase membrane curvature and so facilitate viral budding (Kalvodova *et al.*, 2009), and notably PS enhances the interaction of RV NSP4 with membranes of the endoplasmic reticulum (Huang *et al.*, 2001). It can be speculated that increased membrane curvature of LDs to promote LD plasticity is achieved (in part) through specific upregulation of PS, although lipidomic analysis does not highlight the importance of any particular species of PS.

TAGs were the only lipid class not increased during RV infection. When there are high energy demands on the cell, TAGs are metabolized to fatty acids, which were found to be the most increased class of lipids during RV infection. It is plausible that – as a consequence of RV infection – an upregulation of TAG production occurs synchronously with its metabolic breakdown (lipolysis).

To determine whether LDs increase in size during RV infection, the [phospholipid: (TAG+CE+CH)] ratios were calculated, assuming these to be measures for SA and V, respectively, of LDs. In three out of four analyses, the SA/V ratio decreased, suggesting that the volume of the LDs had increased. In the single experiment where the opposite result was found, it is possible that the treatment with anti-mitochondrial antibody caused the larger LDs to disintegrate. Mitochondria are the site of cellular fatty acid oxidation, and physical associations of these organelles with LDs have been reported (Blanchette-Mackie & Scow, 1983; Kalashnikova & Fadeeva, 2006; Novikoff *et al.*, 1980; Stemberger *et al.*, 1984; Sturmey *et al.*, 2006).

[Phospholipid: (TAG+CE+CH)] ratios were calculated to determine whether RV viroplasms are associated with a specific subpopulation of LDs, but no significant difference was found compared to uninfected cell lysates. Closer inspection of the species of each LD-incorporated lipid class inferred that there was no significant redistribution of particular phospholipid or CE species, suggesting a lack of effect of RV infection on cellular lipid desaturase and elongase activities. The question whether subpopulation-specific LD recruitment by the virus is proteome-specific remains to be explored.

In conclusion, we have demonstrated that total cellular lipid content increases during RV infection and that the lipid increase is consistent with an increase in abundance of LDs interacting with viroplasms. The physiological importance of these changes remains to be defined, with regard to the identification of the components and mechanisms of LD and viroplasm interactions, and the chronology of these events.

## Methods

### 

#### Infection of cells with RV.

MA104 monkey kidney cells were maintained in Dulbecco’s minimal essential medium (DMEM) supplemented with 5 % FCS (Gibco) and antibiotics. The bovine rotavirus RF strain (G6P6[1]) was maintained by passage in MA104 cells as described previously (Arnoldi *et al.*, 2007). Confluent MA104 cells were infected with the RV RF strain at m.o.i. of 5–10 and incubated in DMEM supplemented with 1 µg trypsin IX (Sigma) ml^−1^ for 8 h. Control uninfected cells were processed in parallel under the same conditions.

#### Iodixanol gradient ultracentrifugation of extracts of RV-infected and uninfected cells.

Gradient centrifugation and subcellular fractionation of RV-infected and uninfected cells was undertaken as described previously (Cheung *et al.*, 2010). Briefly, discontinuous iodixanol gradients were performed by layering 1 ml aliquots of 52 %, 42 %, 32 % and 20 % iodixanol in buffered sucrose (0.2 M sucrose, 10 mM Tris/HCl, pH 7.4) on top of one another in 5 ml Ultra-Clear centrifuge tubes (Beckman). Cells were lysed at 8 h post-infection in 2 ml hypotonic lysis buffer (50 mM HEPES, 2 mM MgCl_2_, 1 mM EDTA, pH 7.4) supplemented with protease inhibitors (Roche) by mechanical scraping as described (Miyanari *et al.*, 2007). Cell lysates were gently homogenized by 30 strokes of Dounce homogenization. Half of the sample was then incubated in the presence of Protein A Sepharose A beads (GE Healthcare) which had been conjugated with anti-mitochondrial antibodies (ab3298, Abcam) in accordance with the manufacturer’s protocol to deplete these lipid-rich cellular organelles. After 1 ml of each sample had been placed on 4 ml of an iodixanol gradient, gradients were centrifuged for 16 h at 79 000 *g* and 4 °C, using an SW40 rotor in a Beckman L8-70 centrifuge, and 0.4 ml fractions were collected from the top. The density of the fractions was determined by refractometry.

#### RV RNA extraction and analysis on agarose gels.

This procedure was carried out as described previously (Cheung *et al.*, 2010).

#### Western blotting.

Western blotting was carried out following established protocols (Arnoldi *et al.*, 2007; Cheung *et al.*, 2010). Wet transfer was undertaken overnight at 30 V and 4 °C. Membrane strips were incubated with primary antibodies against the proteins of interest for 4 h in sterile filtered 5 % milk/0.05 % Tween at room temperature (ADRP antibody, Abcam ab52355; and VP1 antibody, produced in guinea pigs and kindly provided by John Patton, NIAID, NIH, Bethesda MD 20892, USA). Membranes were incubated with the appropriate anti-species HRP conjugated secondary antibodies in sterile filtered 5 % milk/0.05 % Tween (anti-rabbit CAT or anti-guinea pig CAT). Membranes were developed using Amersham ECL Plus Western blotting Detection Reagents (GE Healthcare). The blots were then exposed to medical X-ray film (Fujifilm) for 5 s to 2 min according to the intensity of signal.

#### Mass spectrometry.

Each gradient fraction obtained by iodixanol gradient ultracentrifugation was spiked with appropriate internal standards, supplied by Avanti Polar Lipids (100 ng 17 : 0-CE, 200 ng D7-CH, 50 ng 12 : 0/12 : 0/12 : 0-TG, 200 ng 12 : 0/12 : 0-DG, 200 ng 12 : 0-MG, 200 ng 17 : 0-FA, 50 ng C17-Cer, 50 ng C17-SG, 200 ng 14 : 0/14 : 0/14 : 0/14 : 0-CL, 50 ng 12 : 0/12 : 0-PG, 100 ng 12 : 0/12 : 0-PE, 100 ng 12 : 0/12 : 0-PS, 100 ng 17 : 0/20 : 4-PI, 100 ng 12 : 0/12 : 0-PA, 100 ng 12 : 0/12 : 0-PC, 100 ng 17 : 0-LPA, 100 ng 17 : 0-LPC, 100 ng 12 : 0-Cer1P, 100 ng C17-S1P, 50 ng C17-SM and 100 ng C17-SPC), and extracted with a modified Folch method (Folch *et al.*, 1957). In brief, the gradient fractions were extracted with 4 ml chloroform:2 ml methanol:2 ml 0.88 % NaCl [a 2 : 1:1 (v:v:v) ratio]. Then the upper phase was re-extracted with 3 ml of a mixture of chloroform:methanol:0.88 % NaCl as above. The combined lower phase lipid extracts were dried in a Thermo SpeedVac at room temperature and redissolved in 50 µl chloroform/methanol 1 : 1, and 7 µl were injected onto a silica gel column for LC/MS/MS analysis. A Shimadzu IT-TOF LC/MS/MS system, hyphenated with a five-channel online degasser, four-pump, column oven and autosampler with cooler Prominence HPLC (Shimadzu) was used for lipid analysis.

In detail, lipid classes were separated on a normal phase silica gel column (2.1×150 mm, 4micro, MicroSolv Technology) with hexane/dichloromethane/chloroform/methanol/acetonitrile/water/ethylamine solvent gradients (Rainero *et al.*, 2012) based on the polarity of the head group. Accurate mass (mass accuracy of ~5 p.p.m.) and tandem MS were used for molecular species identification and quantification. The identity of lipids was further confirmed by using appropriate lipid standards. The IT-TOF MS operation conditions were: ESI interface voltage +4.5 kV for positive ESI and −4 kV for negative ESI, heat block temperature 230 °C, nebulizing gas flow 1.4 l min^−1^, CDL temperature 210 °C, with drying gas on at pressure of 100 kPa. All the solvents used for lipid extraction and LC/MS/MS analysis were of LC-MS grade, obtained from Fisher Scientific.

For cholesterol (CH) and cholesterol ester (CE) analysis we modified the method of Liebisch *et al.* (2006). Briefly, lipid extracts remaining from the other lipid analyses were dried at room temperature using a Thermo Speed Vac. The dried lipid residues were acetylated with 170 µl acetyl chloride/chloroform [ratio (v/v) 1 : 5] at room temperature for 2 h. The acetylation solvent was removed at room temperature under vacuum. The acetylated residues were redissolved in 40 µl 10 mM ammonium acetate in methanol/chloroform 3 : 1 (v/v), and 7 µl were injected via autosampler for ESI-MS/MS analysis. Shimadzu Prominence HPLC hyphenated with ABSciex 4000 QTRAP was used for CH and CE analysis; 0.25 ml min^−1^ of 7.5 mM ammonium acetate in methanol/chloroform 3 : 1 (v/v) was pumped to the 4000 QTRAP source for ESI-MS/MS analysis. The following MS operation parameters were used: (i) source/gas parameters: curtain gas: 20; collision gas: medium; ion spray voltage: 5500 V; temperature: 400 °C; ion source gas 1 : 45; ion source gas 2 : 20; interface heater: on; (ii) compound parameters: entrance potential: 9.0; collision cell exit potential: 11.0; declustering potential: 60 for CE analysis, 50 for acetylated CH analysis; collision energy: 19 for CE analysis, 15 for acetylated CH analysis.

Both Q1 and Q3 mass were set at unit resolution for multiple reaction monitoring (MRM) analysis of each molecular species of CE and CH. For the detailed MRM setup reference should be made to Liebisch *et al.* (2006).

The experimental steps undertaken during this work are summarized in Fig. S1.

#### Statistics.

Lipid concentrations were compared by paired *t*-test; *P* values of <0.05 were considered to be significant.
